# An attention-based deep learning for acute lymphoblastic leukemia classification

**DOI:** 10.1038/s41598-024-67826-9

**Published:** 2024-07-29

**Authors:** Malathy Jawahar, L. Jani Anbarasi, Sathiya Narayanan, Amir H. Gandomi

**Affiliations:** 1 Leather Process Technology Division, CSIR-Central Leather Research Institute, Chennai, India; 2 School of Computer Science and Engineering, Vellore Institute of Technology, Chennai, India; 3 School of Electronics Engineering, Vellore Institute of Technology, Chennai, India; 4 Faculty of Engineering and Information Technology, University of Technology Sydney, Sydney, NSW 2007 Australia; 5 University Research and Innovation Center (EKIK), Óbuda University, 1034 Budapest, Hungary

**Keywords:** Convolutional neural network, Attention layer, Computer-aided diagnosis, Deep learning models, Leukemia, Computational science, Computer science, Machine learning

## Abstract

The bone marrow overproduces immature cells in the malignancy known as Acute Lymphoblastic Leukemia (ALL). In the United States, about 6500 occurrences of ALL are diagnosed each year in both children and adults, comprising nearly 25% of pediatric cancer cases. Recently, many computer-assisted diagnosis (CAD) systems have been proposed to aid hematologists in reducing workload, providing correct results, and managing enormous volumes of data. Traditional CAD systems rely on hematologists’ expertise, specialized features, and subject knowledge. Utilizing early detection of ALL can aid radiologists and doctors in making medical decisions. In this study, Deep Dilated Residual Convolutional Neural Network (DDRNet) is presented for the classification of blood cell images, focusing on eosinophils, lymphocytes, monocytes, and neutrophils. To tackle challenges like vanishing gradients and enhance feature extraction, the model incorporates Deep Residual Dilated Blocks (DRDB) for faster convergence. Conventional residual blocks are strategically placed between layers to preserve original information and extract general feature maps. Global and Local Feature Enhancement Blocks (GLFEB) balance weak contributions from shallow layers for improved feature normalization. The global feature from the initial convolution layer, when combined with GLFEB-processed features, reinforces classification representations. The Tanh function introduces non-linearity. A Channel and Spatial Attention Block (CSAB) is integrated into the neural network to emphasize or minimize specific feature channels, while fully connected layers transform the data. The use of a sigmoid activation function concentrates on relevant features for multiclass lymphoblastic leukemia classification The model was analyzed with Kaggle dataset (16,249 images) categorized into four classes, with a training and testing ratio of 80:20. Experimental results showed that DRDB, GLFEB and CSAB blocks’ feature discrimination ability boosted the DDRNet model F1 score to 0.96 with minimal computational complexity and optimum classification accuracy of 99.86% and 91.98% for training and testing data. The DDRNet model stands out from existing methods due to its high testing accuracy of 91.98%, F1 score of 0.96, minimal computational complexity, and enhanced feature discrimination ability. The strategic combination of these blocks (DRDB, GLFEB, and CSAB) are designed to address specific challenges in the classification process, leading to improved discrimination of features crucial for accurate multi-class blood cell image identification. Their effective integration within the model contributes to the superior performance of DDRNet.

## Introduction

Leukocytes, also known as white blood cells, assist the immunological system in preventing infections from diseases caused by viruses, fungi, and bacteria. Blood cells called leukocytes are generated in the bone marrow and spread throughout the body. The excessive production of immature lymphocytes in the bone marrow due to acute lymphoblastic leukemia can be fatal, posing a significant risk of mortality among both children and adults. By identifying stained microscopic cell subtypes subjectively, a pathologist does manual categorization. The Kimura stain is used to stain cells, which colors nuclei blue and makes it simpler to identify them. Manual differentiation is based on cell size, transparency, granularity, and nucleus structure. However, there is a lot of variety in cell shape, even within subtypes of the same cell. Therefore, the effectiveness of manual classification depends on the pathologist’s training and expertise. Contrarily, automatic classifications are based on each cell’s physical and chemical properties and procedures^1^.

Blood is composed of blood cells and plasma, with blood cells accounting for around 45% of the total volume and plasma making up the remaining 55%. Erythrocytes, leukocytes, and platelets are the three types of blood cells. Red blood cells constitute around 40–45% of the blood, while white blood cells make up only about 1%^2^. The five types of white blood cells are eosinophils, lymphocytes, monocytes, neutrophils and basophils. The two main subtypes of these blood cells are nongranulocytes (agranulocytes and granulocytes^3^. Eosinophils, neutrophils, and basophils refer to the class granulocytes, whereas lymphocytes and monocytes belong to the class agranulocytes. The five main categories of white blood cells each serve a particular purpose and provide information about the patient’s health in various ways (subjects). Therefore, differentiation of the various types of white blood cells can be an intriguing task. In particular, accurate identification makes it possible to count the various white blood cells and determine if they are present in the right or conventional measure. Moreover, different types of white blood cells can be isolated once identified and thoroughly evaluated for any abnormalities. Analyzing white blood cells offers both quantitative and qualitative insights into a patient’s health. For example, it is possible to diagnose patients with immune system disorders, malignant cells, and leukemia diseases^4^ by examining their white blood cells. The process of identifying blood cells often occurs in a laboratory setting. In this process, blood cell images are stained with specific chemicals, also known as reagents, and then examined using a light microscope by pathologists. Due to the delicate nature of the process, there can be no (or very few) inspection mistakes made by human professionals. Unfortunately, due to exhaustion, doctors might fail to identify the various white blood cells after a lengthy examination.

Despite some progress made in leukemia classification using computer-aided diagnosis technology^5–13^, applying artificial intelligence to clinical diagnosis remains challenging due to microscopic blood cells’ complex inter and intra-class variations. Conventional machine learning (ML) and deep learning (DL) algorithms often overlook the potential correlation among granulocyte and agranulocyte feature maps. Traditional deep learning models fail to eliminate unnecessary information from feature maps, while previous convolutional neural network (CNN)-based methods for leukemia detection suffer from poor generalization due to overfitting issues. The CNN architecture’s design, which directs the input feature layer through the intermediate layers and finally maps the output feature layer, is responsible for the model’s ability to differentiate the output class. Expanding the number of convolution layers in a DL architecture can lead to redundant computation memory and higher time consumption. Additionally, deeper CNNs pose a greater risk of gradient disappearance, which makes it challenging to train the network effectively. Furthermore, the repeated use of pooling operations can result in the loss of global and local context feature information.

Conventional deep learning models cease to disregard extraneous facts in feature maps. Moreover, the overfitting problems in CNN-driven leukemia detection methods lead to insufficient generalization ability. The design of CNN architecture that flows the input feature layer into the intermediate layers and finally mapping the output feature layer decides the learning ability of the model to discriminate the output class. Increasing the number of convolution layers in the DL architecture causes redundant computation memory and time consumption. Moreover, the depth of the CNN increases the risk of gradient disappearance and makes it hard to train the network. Global and local context feature information also gets lost due to continuous pooling operations.

Our proposed DDRNet model aims to tackle the challenges mentioned above and enhance the accuracy of acute lymphoblastic leukemia (ALL) classification. The Deep Dilated Residual Convolutional Neural Network (DDRNet) architecture is designed with a Deep Residual Dilated Block (DRDB), an optimized feature block that incorporates dilated convolutions in residual layers. This results in a feature map with a higher degree of nonlinearity and fewer training parameters, leading to the generation of more discriminative features. To boost the network’s proficiency in feature generation, we proposed the use of a Global and Local Feature Enhanced Block (GLFEB) to capture enriched global and local feature maps. An improved Channel and Spatial Attention Block (CSAB) module was also employed to learn the interrelationship between spatial and channel features. The CSAB module uses global max and average pooling with two fully connected layers to highlight important feature channels and suppress irrelevant features. The DDRNet model integrates batch normalization (BN) and dropout (DO) techniques to enhance the stability and performance of ALL classification. These techniques help to maintain a consistent input distribution across various layers and reduce overfitting. The efficiency of the recommended DDRNet model was assessed on a publicly available Kaggle dataset^14^. Experimental results showed that DDRNet outperformed conventional algorithms with respect to computational time and accuracy. and superior generalization performance for multiclass acute lymphoblastic leukemia classification. It is worth noting that the acronym DDRNet is defined with different acronyms in literature and is used in various contexts.

Our main contributions to the DDRNet model are as follows:

- In order to learn accurate classification features, the CNN design architecture employs several deep residual dilated blocks. This architecture’s residual path prevents vanishing gradients during backpropagation, thereby facilitating faster convergence.
- The local and global features obtained from the initial convolution layer are concatenated with the GLFEB feature to improve the classification feature’s representation ability.
- The inter-channel and inter-spatial features are learned and employed by the CSAB to achieve more excellent feature representation.
- During the training and testing of DDRNet custom models, the accuracy and loss metrics are utilized to assess the obtained results.

The paper is organized as follows: In “Related work” section, previous research on the detection of Acute Lymphocytic Leukemia using microscopic images will be reviewed, along with the shortcomings of current methods. Section “Proposed work” describes the proposed DDRNet model and the corresponding detailed architecture. Section “Results and discussion” presents the experimental setup, data augmentation, model evaluation, and performance analysis. Results and discussion are presented in this section. Section “Conclusion” summarizes the findings of the study and explores potential avenues for future research.

## Related work

### Machine learning algorithms

Support vector machine (SVM) and k-nearest neighbor (k-NN) were proposed by Paswan et al.^5^ to categorize the Acute Myeloid Leukemia (AML) leukemia subtype and achieved 83% accuracy. SVM was used by Patel et al.^6^ to classify the blood cell images with 93% accuracy. Karthikeyan et al.^7^ utilized fuzzy C-mean clustering techniques and SVM to distinguish ALL and attained 90% accuracy. Random forest (RF) and the grey-level co-occurrence matrix were utilized by Mishra et al.^8^ to classify ALL. Some estimated characteristics are useless because of the nucleus’s round shape, resulting in a feature matrix with a high computational cost. With the same data, the RF classifier’s accuracy attained 99.004%, which is more than that of Di Ruberto^15^, which is 92%. Shape, color, and texture attributes from ALL images were used by Mohapatra et al.^16^ used an ensemble of classifiers to classify the images. They showed that even though the support vector machine’s specificity value is higher than the EOC classifier’s, the sensitivity and accuracy of the SVM are less than yet similar to those of the EOC classifier. Different SVM kernels^17^ and feature selection techniques based on SVM, neural networks (NN), trees, and RF, among other classifiers, were also utilized for better classification. Results showed that, compared to RF, the SVM classifier produced superior results. Aimi et al.^18^ retrieved 42 attributes, including form, color, and size, which were used to classify the cells using the condensed fuzzy art map and multilayer perceptron (MLP). The results show how significantly the recovered features improved classification accuracy. Mishra et al.^19^ analyzed higher-order discrete cosine transforms coefficients. For the classification, they used many classifiers, including SVM, backpropagation NNs, NN, and KNN classifiers. They demonstrated that SVM outperformed the other classifiers in specificity and accuracy. Neoh et al.^20^ proposed a new segmentation technique using stimulating discriminant measures and extracted 80 features retrieved from the segmented nucleus and cytoplasm, such as color, texture, and geometry. They then examined the features using a different classifier and achieved an accuracy of 96.72%. Rawat et al.^21^ initially extracted 142 features, such as color, texture, and shape, to build a hierarchical classifier. They then employed artificial NN fuzzy inference systems, KNN, probabilistic SVM, and NN classifiers. The drawbacks of this research include the heavy computational load, the extensive usage of features, and the egregious errors in reporting the results. Abbasi et al.^22^ extracted features from nucleus images using the k-means and watershed algorithms. The authors used the support vector machine approach to classify the images into binary and multiclass. Also, principal component analysis (PCA) reduced the dimension of the feature space to avoid overfitting and attained 99% accuracy, 99% specificity, and 97% sensitivity. ALL and AML are the two forms of leukemia, and Reta et al.^23^ have suggested a model to classify them. Blood cell segmentation is accomplished through the utilization of contextual color and texture data, which aids in the segmentation of overlapping blood cells to identify the nucleus and cytoplasm. After segmentation, several machine learning classifiers offered in Weka were utilised to extract morphological, statistical, texture, size ratio, and eigenvalues characteristics^3^. Ravikumar et al.^24^ separated white blood cells from the acquired images using k-means clustering following feature extraction, feature selection using PCA, and artificial neural network classification. The Fast Relevance Vector Machine (F-RVM), a technique for segmenting and classifying white blood cells, was proposed in^25^. Portions of white blood cells were separated using Otsu’s thresholding method. Elements unrelated to white blood cells were removed using mathematical morphological techniques. The Naive Bayes classifier was trained using the features extracted from the segmented cell nucleus.

### Deep learning approaches

Deep neural networks have enabled us to do better in image vision. Deep neural networks are frequently used to analyze medical images for categorizing diseases, localization, detection, registration, and segmentation^26–28^. However, the size of the dataset affects how well a deep neural network performs, where obtaining a vast dataset for medical analysis is challenging. (ViT-CNN)^9^ classifies images to diagnose acute lymphoblastic leukemia and achieved an accuracy of 99.03%, and experimental comparisons show that it outperforms other models. Convolutional neural network deep learning techniques and reliable segmentation are utilized for training the model using bone marrow images, yielding a classification accuracy of 97.78%^10^ for private data. Das et al.^11^ proposed a quicker and more efficient method to analyze Acute Lymphoblastic Leukemia (ALL), a blood malignancy, employing depth-wise separable linear convolutions architecture with inverted and skip residual connections. The proposed plan includes a probability-based factor that successfully combines MobilenetV2 and ResNet18 while maintaining the merits of both approaches. Using the available benchmark datasets ALLIDB1 and ALLIDB2, its performance is verified. The experimental findings show that the suggested technique results in the best accuracy in the ALLIDB1 and ALLIDB2 datasets, 99.39% and 97.18% (with 70% training and 30% testing), respectively.

To obtain higher-quality in-depth features from the images, Ullah et al.^29^ analyzed a CNN-based model to identify the visual geometry of VGG16 and the Efficient Channel Attention (ECA) model. The proposed model has improved the feature representation and classification performance by utilizing contextual color and texture data to segment overlapping blood cells. The use of the ECA module has effectively reduced the morphological similarities between ALL cancerous and healthy cell images. Duggal et al. proposed CNN model that extracted deep features with 91.1% accuracy on the C-NMC 2019 dataset^30^. An automated diagnostic method was presented by Anwar et al.^31^ that uses a convolutional neural network (CNN) model to identify acute lymphoblastic leukemia (ALL). The model is evaluated on 221 test images, which achieved accuracy levels of nearly 100% during training and 99.5% testing accuracy. A fivefold validation approach was used to train the model using 515 images, resulting in an accuracy of 95.54%. The method effectively uses unprocessed data without the requirement for pre-processing or segmentation. As a result, this technique may help pathologists diagnose ALL effectively. The automated system uses^32^ an object identification technique that can tell whether leukemic cells are present from tiny blood smear images. For cell detection and cell classification, the You Only Look Once (YOLOv4) method version 4 was used. As a result, each image was classified as blast cells (ALL) or healthy cells., and a binary problem was set up for the classification (HEM). The image analysis program was trained and tested on the ALL-IDB1 and C-NMC 2019 datasets. The mAP (Mean Average Precision) achieved for the ALL-IDB1 dataset was 96.06%, while the C-NMC 2019 dataset attained an accuracy of 98.7%. Malathy et al.^33^ proposed an ALNett, a depth-wise convolution model enhanced with dilation rates for classifying binary images of acute lymphocytic leukemia. Convolution, max-pooling, and normalization are the unique cluster layers employed to fetch important global and local information for accurate ALL prediction. These layers also provide additional structural and contextual information attaining 91.13% and 0.96 as accuracy and F1-score.

Without employing image segmentation or feature extraction, which require laborious computations, Anilkumar et al.^12^ classified ALL using Deep Convolutional Neural Networks (DNNs). The classification uses images from the American Society of Haematology’s (ASH) online image repository. Using pre-trained AlexNet and LeukNet, a newly designed deep learning network, the work successfully distinguished the B-cell and T-cell ALL images, achieving a classification accuracy of 94.12%. Granero et al. studied^13^ the usage of quaternion-valued convolutional neural networks and investigated the performance of lymphoblasts from microscopic images of peripheral blood smears. Despite utilizing only 34% of its parameters, the quaternion-valued convolutional neural network outperformed or was at least as effective as its real-valued counterpart. This outcome demonstrates how quaternion algebra enables information to be captured and extracted from a color image with fewer parameters. Machine learning, deep learning methodologies, leukemia detection challenges, and various medical modalities to be analyzed are detailed in these research works^34–36^. Table 1 presents an overview of the machine learning and deep learning architectures employed for the classification of ALL.

**Table 1:** Details of ML and DL architectures for ALL classification.

Reference no	Algorithm/architecture Deployed year	Approach	Drawbacks
^5^	SVM and K-NN (2017)	Machine Learning	Local binary Features were extracted where it failed to detect the overlapping and irregular cells
^6^	SVM (2015)		Feature-based detection where leukemia-infected regions were not detected accurately
^8^	Random Forest (2017)		Excelled in the classification of nucleus and cytoplasm, texture features can be overcome with color and morphological features for better performance. The time complexity is high due to the ensembling technique
^22^	*k*-means and watershed algorithms with PCA (2020)		This method has overcome the overfitting problem through multifractal features but results in time complexity
^9^	ViT-CNN (2021)	Deep Learning	Noise and data balance through vision transformer
^10^	CNN (2018)		Improved the classification for the subtypes using the morphology of L2 and L3 blasts Failed for overlapped cells
^11^	MobileNetV2 + ResNet18 (2021)		Poor performance is achieved for data split of 50% training and testing
^32^	YOLOv4 (2021)		colour fidelity, optimal brightness and contrast, resolution and general artefact reduction of microscopic blood smear images affect the accuracy
^33^	ALNett (2022)		Structural and contextual feature extraction has to be improved to improve accuracy
^12^	AlexNet + LeukNet (2021)		Feature extraction is not performed where the dataset is small
^37^	CNN + GAN (2022)		Data augmentation is required to generate additional instances
^39^	CNN + ML + Transfer Learning (e.g. ResNet50 combined with Random Forest)	Hybrid	Performance depends upon the choice of machine learning algorithm

Ziyue et al.^37^ utilized a combination of Generative Adversarial Networks (GANs) and CNNs to develop a highly accurate and efficient technique for detecting malware. They are achieved by generating additional instances through data augmentation, which can be applied to enhance ALL images. Han et al.^38^ performed a study on an Unmanned Ground Vehicle (UGV) trajectory design method to counteract malicious radio sources, particularly those that are concealed. The study aimed to compare this approach with existing efforts. A novel deep learning framework, utilizing the internet of health things (IoHT), has been proposed to precisely identify and categorize cervical cancer in Pap smear images. This framework combines a hybrid CNN, machine learning, and transfer learning models to generate the necessary features for accurately classifying normal and pathological cervical cells^39^.

Abhishek et al.^40^ presented a new dataset of 750 microscopic blood images depicting different forms of leukemia, such as chronic lymphocytic, acute lymphoblastic, chronic myeloid, and acute myeloid leukemia. The purpose of this dataset is to facilitate the detection and classification of leukemia using deep transfer learning techniques. In addition, the study assesses the durability of trained models using test datasets that are not specific to any particular subject. It also examines classification results in both qualitative and quantitative terms, and uses class-specific heatmaps to visually represent distinguishing features. Arif et al.^41^ proposed a new technique to improve leukemia classification by utilizing the MobileNetV2 architecture. They achieved this by fine-tuning the architecture on a large dataset of leukemia images and incorporating global average pooling after transfer learning. This strategy aims to decrease the dimensionality of spatial data in transfer learning weights and extract consistent information from each feature map. Mortez et al.^42^ conducted a study using deep neural networks to eliminate the need for feature extraction in ALL classification. The convolutional neural network achieves a remarkable accuracy of around 97% in categorizing six ALL and lymphocyte subtypes.

Kazeminia et al.^43^ introduced a method called Multiple Instance Learning (MIL) to tackle the issue of poorly labelled data. This approach utilizes powerful encoders that are usually trained with labelled data. This work addresses the use of Self-Supervised Learning (SSL) in combination with multihead attention to categorize the subtype classification from blood smears. Gokulkannan et al.^44^ presented a highly effective model for detecting leukaemia. The model utilizes the Multiscale Trans-Res-Unet3 + Network to segment the region of interest. The Election-Based Chameleon Swarm Algorithm (E-CSA) is utilized to extract and improve relevant parameters such as colour, shape, and texture. The recommended leukaemia detection model’s performance rate is analysed using a Multiscale Adaptive and Attention-based Dilated Convolutional Neural Network. This analysis is done by comparing it with conventional leukaemia detection models and existing algorithms. Various performance metrics are used for validation. Siraj et al.^45^ utilized a deep convolutional generative adversarial network (DCGAN) and a dual attention method to accurately classify peripheral blood cell (PBC), leukocyte images for segmentation and classification (LISC), and Raabin-WBC benchmark datasets. They achieved an overall accuracy of 99.83%, 99.35%, and 99.60% respectively.

Weizheng et al.^46^ introduced a robust authentication protocol for Wireless Multimedia Sensor Networks (WMSN) by utilizing blockchain technology, Physically Unclonable Functions (PUFs), and a fuzzy extractor technique for biometric data. This system undergoes thorough security examination and exhibits superior performance in terms of computation and communication cost when compared to existing protocols. Sharnil et al.^47^ utilized the “Cardiac-200” dataset and the benchmark dataset “PhysioNet” to examine and categorize different acoustic occurrences of heartbeats. They achieved notable enhancements in accuracy by utilizing the proposed InfusedHeart Framework. The framework achieved an accuracy of 89.36% (without augmentation) and 93.38% (with augmentation), surpassing previous ML and DL techniques and demonstrating the potential for further advances in cardiac acoustic event classification. Table 1 summarizes the details of ML and DL architectures deployed for ALL classification.

## Proposed work

This section introduces a novel Dilated Deep Residual Convolution Neural network (DDRNet) for blood cell classification (ALL). DDRNet is composed of deep residual dilated block optimized feature Block (DRDB), a global and local feature enhanced block (GLFEB), and Channel and Spatial Attention Block (CSAB). The DRDB uses standard and dilated convolution to exchange the receptive size to capture improved features. The GLFEB enhances the identification of expressive features by integrating local and global features in blood cell classification. The CSAB block learns features from the different channels of a multi-channel input as well as the various spatial locations within the blood cell image. DRDB, CSAB and GLFEB block features provide an excellent representation of features for blood cell classification. Feature map regularization is achieved by adopting dropout in the residual path.

### DDRNet neural network architecture

**Figure 1:**
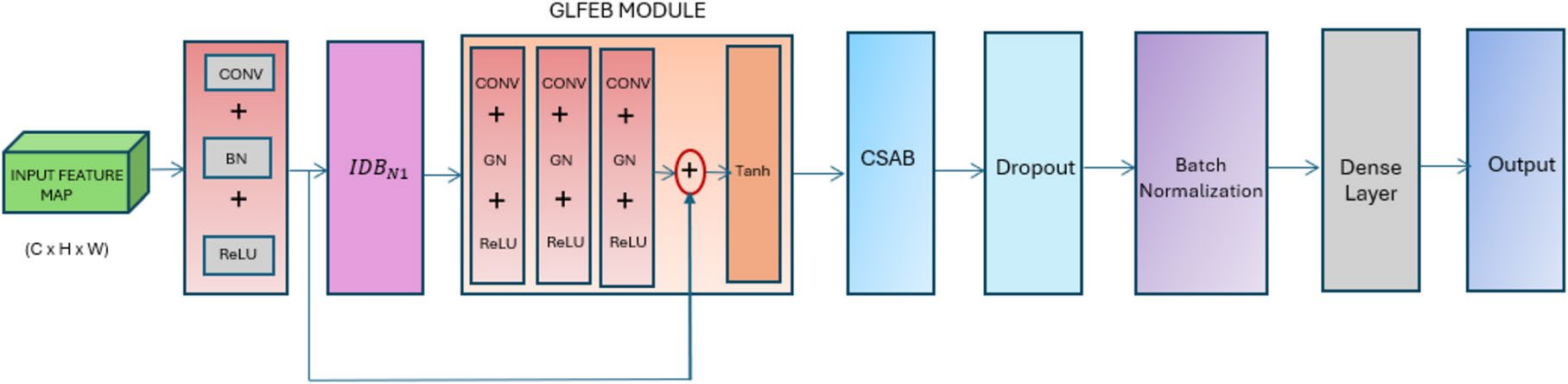
Proposed DDRNet neural network architecture.

The proposed DDRNet layer includes DRDB, GLFEB, and CSAB block, as shown in Fig. 1. DRDB consists of a 6-layer residual block enhancing the performance and effectiveness in image classification by detecting the crucial optimized features. The learning is enhanced due to the optimisation of the optimal mapping and residual mapping, where the residual dilated block is shown in Eq. (1).

1 $$O_{RDB} = f_{RDB} \left( {O_{CN} } \right)$$

The input image processed for classification $f_{RDB}$ is the function representing the DRDB. The input is fed to the convolutional layer followed by group convolution, max-pooling, and ReLU optimization function

2 $$O_{CN} = f_{CN} \left( {I_{N} } \right)$$

$O_{CN}$ is the output of this convolutional layer, where $f_{CN}$ is the function that represents the convolution layer, and $I_{N}$ means the input data (Eq. 2). The three-layer GLFEB retrieves the global features and local optimized residual features, where $I_{N}$ refers to the global and $O_{RDB}$ local features. The implementation is represented as follows (Eq. 3)

3 $$O_{CLFEB} = f_{CLFEB} = (I_{N} , O_{RDB} )$$

wre $O_{RDB}$. represents the output of residual dilated block and $O_{CLFEB}$. represents the output of GLFEB, respectively. This normalized feature is fed to the CSAB for feature refinement (Eq. 4).

4 $${\text{OCSAB = fCSAB (OCLFEB)}}$$

This resulting output is summed with the output from the convolution of GLFEB using element-wise summation, resulting in attention-enhanced features. This resulting feature is normalized using batch normalization along with the dropout layer, resulting in optimized features that generate good accuracy in image classification.

### Convolution block

The convolution block receives the input blood cell images. The convolution layer’s output feature is fed to the max pooling layer, followed by group normalization. Group normalization divides the channel into groups and normalizes the features of each group. The mean and variance computed for normalization is given in Eqs. (5, 6 and 7)

5 $$Mean \left( {\mu_{i} } \right) = \frac{1}{m} \mathop \sum \limits_{{k \in S_{i} }} x_{k}$$

6 $$Variance \left( {\sigma_{i}^{2} } \right) = \frac{1}{m} \mathop \sum \limits_{{k \in S_{i} }} (x_{k} - \mu_{i} )^{2}$$

7 $$feature \left( {\widehat{{x_{i} }}} \right) = \frac{{x_{i} - \mu_{i} }}{{\sqrt {\sigma_{i}^{2} + \in } }}$$

$x_{i}$ is the feature computed by a layer. The features obtained after max pooling are passed through group normalization, which enhances stability and consistency across various batch sizes and data distributions. This normalization technique aids in regularization during training, effectively mitigating overfitting by minimizing internal covariate shift. The group normalization is defined to be 32 in this proposed model. By optimizing the features that have been normalized within a group, using rectified linear unit (ReLU) activation, the network is able to perform nonlinear transformations. These nonlinear transformations allow the model to learn intricate features and patterns necessary for accurate leukemia detection and classification. The utilization of this activation function brings about both sparsity and nonlinearity, which in turn facilitates more effective gradient propagation during the training process and improves the model’s capacity to generate global features and patterns within the data. The resulting feature is fed to the residual dilated block.

### Deep residual dilated block

The DRDB block is a powerful architectural component that improves feature extraction, gradient flow, convergence speed, and generalization abilities in convolutional neural networks by fusing the benefits of residual connections, dilated convolutions, and concatenation. The DRDB block is responsible for capturing multiscale characteristics and addressing the vanishing gradient problem during training. Multiple DRDB blocks are integrated into the model’s architecture. Each DRDB block consists of residual convolution paths and residual dilated convolution blocks. These paths facilitate the extraction of multiscale features, allowing the model to capture intricate details present in blood cell images. Additionally, the residual connections within the DRDB block help mitigate the vanishing gradient problem, enabling more effective training of the model.

**Figure 2:**
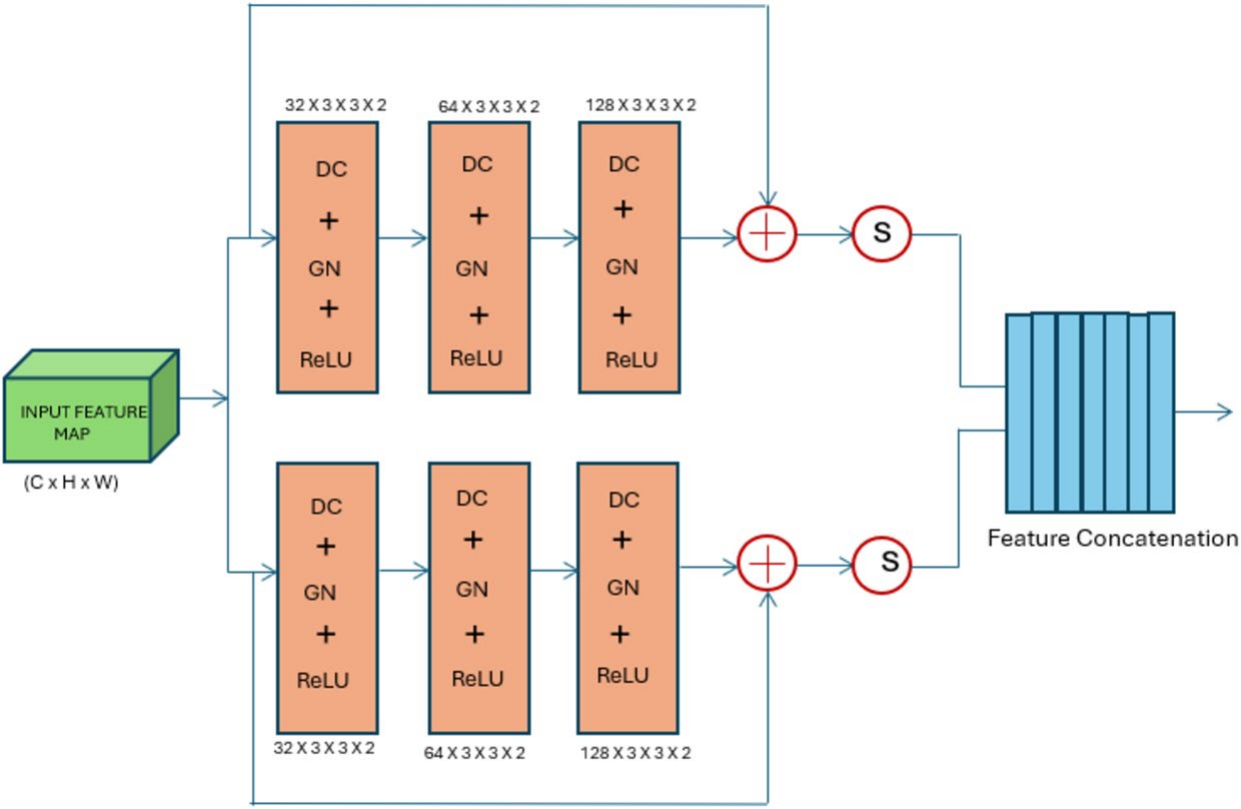
Deep residual dilated block (DRDB).

This block includes six blocks that generate six features and are concatenated before the final convolution layer, as shown in Fig. 2. The DRDB block included three residual convolution paths along with three residual dilated convolution blocks. The outputs are concatenated for further processing. The residual path benefits from accelerating the convergence by evading the vanishing gradient during the back propagation. The residual convolution included convolution, Group normalization, ReLU, convolution and Group normalization. The conventional residual block between the layers is used to retain more original information and extract the general feature maps. Unlike the traditional residual blocks, say those in ResNet50, the DRDB block is capable of extracting sophisticated semantic features and multiscale characteristics, and in terms of gradient flow, the DRDB block addresses the vanishing gradient problem, thereby expediting the convergence. The residual dilated convolution block (2, 4, and 6) had dilated convolution, Group normalization, ReLU, dilated, and Group normalization. The dilate sequence is 1, 2, and 4 in this proposed work. The dilation difference is to capture the information that is not captured by expanding the receptive area without increasing the number of convolution layers. Thus, without increasing the computation complexity, DRDB block employs residual dilated convolution blocks between adjacent layers, making the training much easier and generalization much better.

### Global and local feature enhancement block

The GLFEB block is an essential part that helps deep neural networks represent more data by efficiently capturing both local and global features. GLFEB blocks are inserted after the DRDB blocks in the model’s architecture. These blocks include convolutional layers followed by global average pooling and max-pooling operations. The global average pooling operation captures global features, while the subsequent convolutional layers capture local features. By combining both global and local features, the GLFEB block facilitates more discriminative feature learning, enhancing the model’s ability to differentiate between different blood cell types.

**Figure 3:**
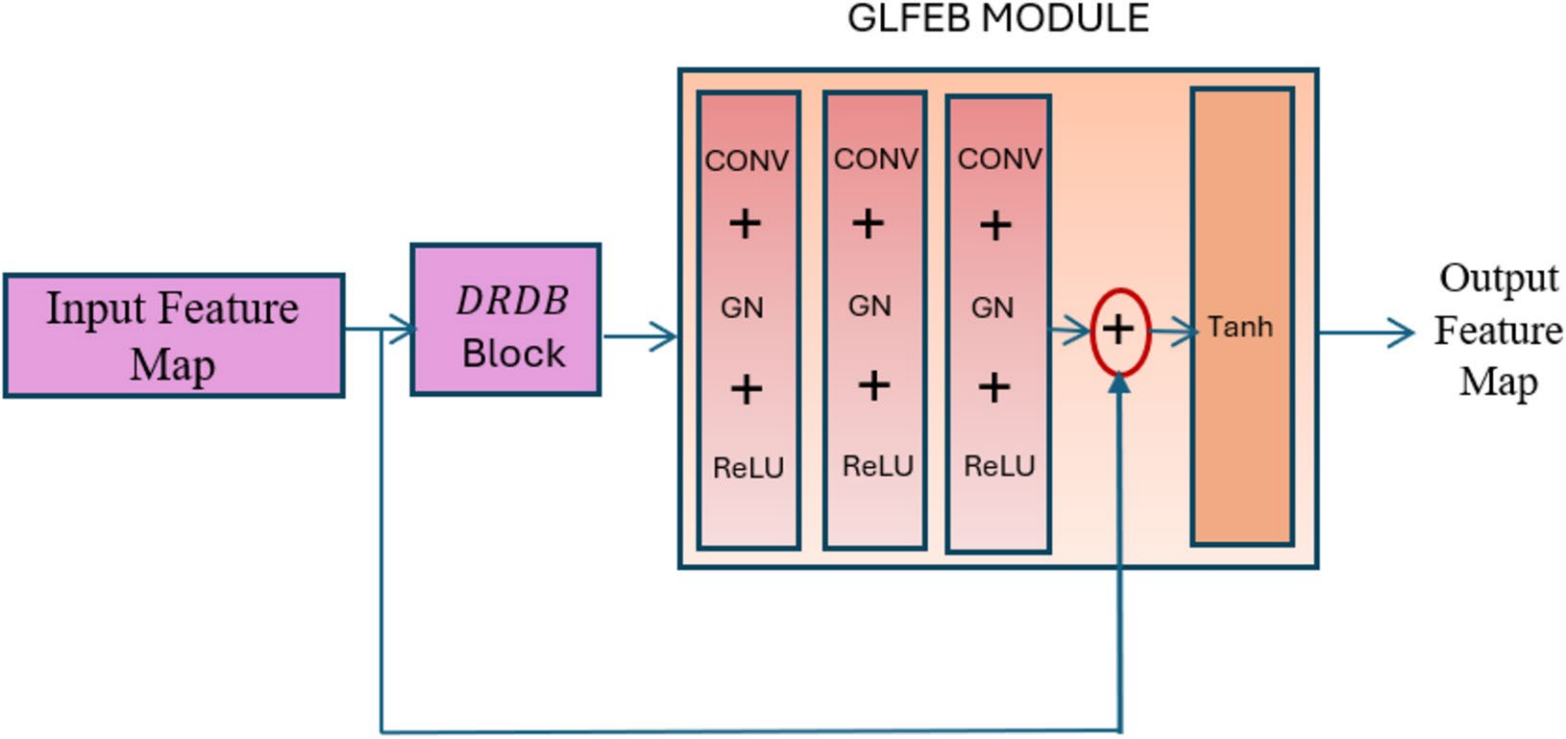
Global and local feature enhancement block (GLFEB).

GLFEB is proposed to overcome issues related to the weakened influences ^48^ that occur in deep networks created from the shallow layers as shown in Fig. 3. GLFEB includes three layers of convolution + Group normalization + ReLU with a filter size of 64 × 3 × 3 × 64 and 128 × 3 × 3 × 128. This is followed by convolution with a filter of 64 × 3x3xc where ‘c’ is similar to the previous layer. The global feature obtained from the initial convolution layer is concatenated with this feature to improve the ability to represent the classification feature. By leveraging both convolutional operations and group normalization, the network can effectively capture spatial dependencies and pattern facilitating more discriminative feature learning, resulting to higher classification accuracy. this block results in robust and efficient features offering improved training stability, regularization, gradient propagation, and feature learning capabilities. The final output is 64 × 3 × 3 × 2c. Tanh function is used to convert the feature into non-linearity as shown in Eq. (8)

8 $$\tanh \left( {F_{GL} } \right) = \frac{{e^{{F_{GL} }} - e^{{ - F_{GL} }} }}{{e^{{F_{GL} }} + e^{{ - F_{GL} }} }}$$

where the function maps the input feature $F_{GL}$ to a range of-1 to 1 exhibiting a symmetric curve about the origin, enhancing the learning process by alleviating the vanishing gradient, resulting in faster convergence.

### Channel and spatial attention block

The CSAB block selectively emphasizes informative features within the input feature maps by capturing inter-channel and inter-spatial relationships. CSAB allow neural networks to dynamically allocate resources to essential features, improving their ability to capture relevant information from input blood cell data. CSAB blocks are integrated towards the end of the model’s architecture. These blocks incorporate both channel attention and spatial attention mechanisms. The channel attention mechanism learns inter-channel relationships, while the spatial attention mechanism captures spatial dependencies within the input data. Enhancing feature discrimination and improving the model’s accuracy in leukemia detection and classification, the CSAB block selectively highlights important features while suppressing noise and irrelevant information.

#### Channel attention mechanism

##### Global average pooling (GAP)

The first step involves applying GAP to the input feature map (At). GAP computes the average value of each channel across all spatial locations (HxW). The result is a vector of size C (the number of channels), where each element represents the average activation of a channel.

##### Linear transformation and ReLU activation

The vector obtained from GAP undergoes a linear transformation followed by a ReLU activation function. This transformation allows the network to learn channel-wise attention weights. These weights emphasize important channels while suppressing less relevant ones.

##### Fully connected layers and sigmoid normalization

The channel-wise feature map ($Q_{c}$) obtained from the ReLU activation is further processed through fully connected layers. These layers are concatenated and fed to a sigmoid function, which normalizes the features. This normalization ensures that the attention weights sum up to 1 across all channels, effectively highlighting informative channels.

#### Spatial attention mechanism

##### Convolutional operation

The spatial attention mechanism operates directly on the input feature map (At), focusing on its spatial dimensions (HxW). A convolutional block with a 5 × 5 kernel captures spatial dependencies and patterns within the feature map.

##### Normalization and ReLU activation

The output of the convolutional block is normalized using a sigmoid function, resulting in spatial-wise attention features ($Q_{s}$). These features represent the importance of spatial locations within the feature map. A ReLU activation function is then applied to introduce non-linearity in the attention computation.

##### Combining attention weights

Normalization Across Channels: The spatial attention features ($Q_{s}$) are normalized across all channels. This ensures that the attention weights sum up to 1 across spatial locations.

Combining Channel and Spatial Attention: Finally, the channel-wise attention weights ($Q_{c}$. ) and spatial-wise attention weights ($Q_{s}$) are combined to produce the overall attention map. This combined attention map guides the network in focusing on relevant channels and spatial regions during feature extraction.

**Figure 4:**
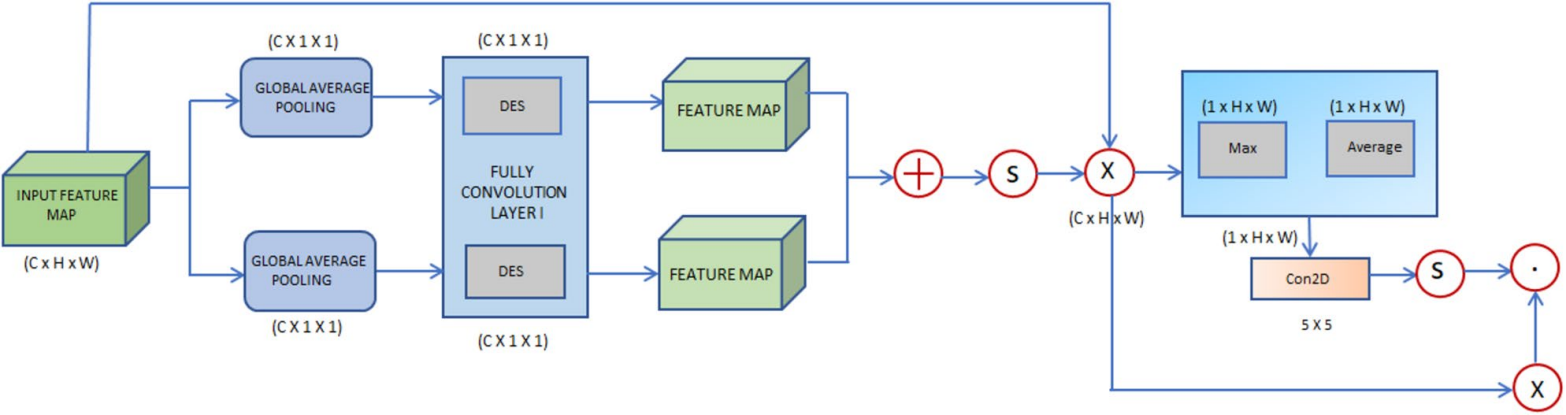
Channel and Spatial Attention Block (CSAB).

The inter-channel and inter-spatial features are learned by the channel and spatial attention block (Fig. 4). After generating the channel attention map, the spatial attention is initially computed from the intermediate feature map. Each feature is multiplied using element-wise computation. Global averaging and max-pooling are employed to achieve more excellent feature representation. The feature map ($$Q_{c} )$$ CxHxW is fed to the global max and average pooling, generating inter-channel features.

e channel attention mechanism computes attention weights for each channel across all spatial locations for global average pooling as given in Eq. (9), where $At$ is the input feature map

9 $$F_{GAP} \left( {At} \right) = \frac{1}{HxW}\mathop \sum \limits_{i = 1}^{H} \mathop \sum \limits_{j = 1}^{W} At_{cij}$$

This feature generated by the global average pooling is transformed linearly, followed by the ReLU activation function to obtain channel wise feature map ($$Q_{c} )$$ as given in Eq. (10).

10 $$(Q_{c} ) = ReLU\left( {W_{1} \cdot F_{GAP} \left( {At} \right)} \right)$$

These are then fed to fully connected layers and are concatenated and fed to the sigmoid function, which normalizes the features generated by the channel attention model. The inter-spatial features ($$Q_{s} )$$ process the CxHxW and generate 1xHxW dimension. This is fed to a convolution block of 5 × 5 kernel and is normalized through the sigmoid function followed by the ReLU activation function given as (11) where $$W_{2}$$ are the learning parameters in convolutional kernel. The obtained spatial attention features are normalied across all the channels and are shown in Eqs. (11 and 12).

11 $$Q_{s} = ReLU\left( {W_{2} *At} \right)$$

12 $$Q_{s} = \frac{{Q_{s} }}{{\mathop \sum \nolimits_{c = 1}^{C} Q_{s} }}$$

The final attention weights are obtained by combining the channel-wise attention features and the normalized spatial-wise attention features. These attention weights are then applied to the input feature map to emphasize informative channels and spatial locations.

A crucial component is the CSAB for deep neural networks to prioritize certain channels and spatial locations within input feature maps. The CSAB improves the network’s feature representation capabilities by combining both channel and spatial attention methods. This improves performance across various tasks, including segmentation, object identification, and classification.

### Significance of the proposed DDRNet model

Accurate classification of blood cell types provides essential information for tailoring treatment plans. Different types of leukemia or blood disorders may require distinct therapeutic approaches, and precise identification enables personalized treatment strategies. Early detection may enable the use of less aggressive treatments, reducing the overall toxicity and side effects associated with more intensive therapies. This is particularly important in enhancing the quality of life for individuals undergoing treatment. Detecting blood cell abnormalities early can prevent the progression of the disease to more advanced and potentially more difficult-to-treat stages. This is particularly relevant for aggressive conditions like lymphoblastic leukemia. The combination of early detection and accurate classification contributes to higher survival rates. Timely intervention and personalized treatment increase the chances of achieving long-term survival.

DDRNet offers a more comprehensive approach by incorporating attention mechanisms (CSAB) alongside feature enhancement (GLFEB) and multiscale feature extraction (DRDB). This integrated architecture leads to better feature representation and discrimination, potentially improving classification accuracy and robustness compared to traditional transfer learning. Its novel architecture tackles important issues in feature extraction and discrimination, indicating that it is a potential model for clinical situations for detection and classification.

The spatial dependencies and patterns of blood cell images are captured by the CSAB blocks’ spatial attention mechanism. This enables the model to recognize significant spatial configurations and combinations of individual cell components, which are frequently suggestive of particular cell types. For example, neutrophils have characteristic multilobed nuclei, whereas lymphocytes usually have a large nucleus-to-cytoplasm ratio. The model efficiently distinguishes between various blood cell types by capturing such spatial dependencies.

The channel attention mechanism in CSAB blocks learns inter-channel interactions within the input feature maps. Different blood cell types may show unique spectral characteristics or intensity distributions in microscope images. Through channel learning, the model extracts and uses relevant spectral features for classification, thereby learning which channels contain the most discriminative information for each cell type.

The retrieved features’ discriminating strength is improved through the usage of the CSAB block by merging the channel and spatial attention methods. This is essential for effectively differentiating between slight variations in the shape and appearance of blood cells that could indicate multiple medical conditions or pathological situations. For instance, the cytoplasm of eosinophils is granular, but the nucleus of monocytes frequently has a kidney shape. The model achieved improved classification accuracy by concentrating on the most pertinent information related to each type of cell.

By drawing attention to the regions or features of the image that have the most influence on the classification decisions, the attention mechanisms also contribute to interpretability. As a result, physicians can better comprehend the rationale behind the model’s predictions and its reasoning process, which can increase the model’s credibility in therapeutic contexts.

## Results and discussion

This section details the image dataset, analysis, DDRNet training, validation, and testing. A comparison of the proposed methodology with previous research studies is also provided for a better understanding.

### Experimental setup of the analysis of DDRNet

In this study, the proposed DDRNet is experimented using a 64-bit Windows 10 operating system. Table 2 lists the computer configuration used for the DDRNet model training and testing. The proposed DDRNet architecture design and implementation were carried out using the deep network designer application of MATLAB 2022 academic version deep learning toolbox 14.4.

**Table 2:** Computer configuration of the proposed DDRNet.

Item	Configuration
Processor	Intel (R) Xeon (R) Gold 6230 CPU @2.10GHz
Graphics card	NVIDIA Quadro RTX 5000 16 GB
Ram size	64 GB
Hard-disk size	2 TB

### Dataset

**Figure 5:**
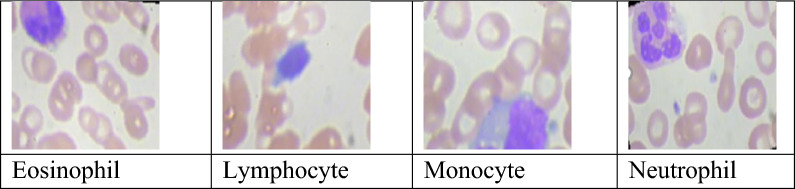
Sample images^14^ .

The experimental analysis was performed through the publicly available database^14^ from the Kaggle website. The dataset includes 362 images of five types: Basophil, Eosinophil, Lymphocyte, Monocyte, and Neutrophil. Each variety contains images of the count, three images of class Basophil, 80 images of class Eosinophil, 35 images of class Lymphocyte, 25 images of class Monocyte, and 220 images of class Neutrophil, as shown in Fig. 5.

### Data augmentation

For easier processing, dataset images were converted to jpeg format and resized to 224 × 224x3. The images were divided into training, validation, and test sets before augmentation was performed. Augmentation techniques were used during the data preprocessing stage to handle class imbalances, especially for under-presented cell types. The augmentation process involves horizontal flipping, rotation to 10 degrees, zooming with a scale of 2, and random contrast enhancement. Since cropping might result in losing some significant information, cropping has not been performed in this work. This augmentation strategy enables the generation of a more balanced and diverse dataset, facilitating effective training and evaluation of DDRNet across all cell types.

Horizontal flipped image $I_{HF}$ is generated from original Image $I_{o}$ by flipping the image horizontally referring to image width ‘$w^{\prime}$ and the transformation is given as Eq. (13).

13 $$I_{HF} \left( {m,n} \right) = I_{o} (w - (m,n))$$

When the model sees both original and flipped images during training, it becomes more adept at recognizing leukemic abnormalities regardless of their orientation in real-world diagnostic scenarios. This helps the model learn robust features that are invariant to horizontal orientation.

Rotated image $I_{RO}$ are generated by rotating the original image $I_{o}$ to 10 degree clockwise and the transformation is shown in Eq. (14), where is $\theta$ the rotation angle in radians (10 degrees converted to radians), and m,n are the coordinates of the pixel in the original image $I_{o}$.

14 $$I_{RO} \left( {m,n} \right) = I_{o} \left( {\cos \left( \theta \right)m + \sin \left( \theta \right)n, - \sin \left( \theta \right)x + \cos \left( \theta \right)y} \right)$$

When the model encounters rotated images during training, it becomes better at handling real-world scenarios where cell structures may appear at different angles. For example, a rotated leukemic cell image should still exhibit recognizable patterns. Rotated images help the model learn features that are invariant to rotation.

Zoomed image $I_{ZO}$ is generated by zooming the image to a scale of 2and the transformation is given in Eq. (15) where m and n are the coordinated of the pixels in the original image $I_{o}$.

15 $$I_{ZO} \left( {m,n } \right) = I_{o} \left( {\frac{m}{2},\frac{n}{2}} \right)$$

Zoomed images allow the model to learn features that remain consistent across different magnifications. Real-world blood cell images contain cells of varying sizes. Augmenting the dataset with zoomed images ensures the model adapts to different cell scales.

Random contrast enhancement increases the contrast of the image in a random manner. This process entails modifying the pixel values to amplify the disparity between the brightest and darkest components in the image, thus enhancing its overall contrast. Clinical blood cell images exhibit diverse contrast levels due to variations in sample preparation and imaging techniques. Varying contrast levels simulate different imaging conditions (e.g., variations in staining, lighting, or equipment settings). The model learns to recognize blood cells even when the lighting varies significantly. By augmenting the dataset with contrast-enhanced images, the model becomes more resilient to such variations.

**Figure 6:**
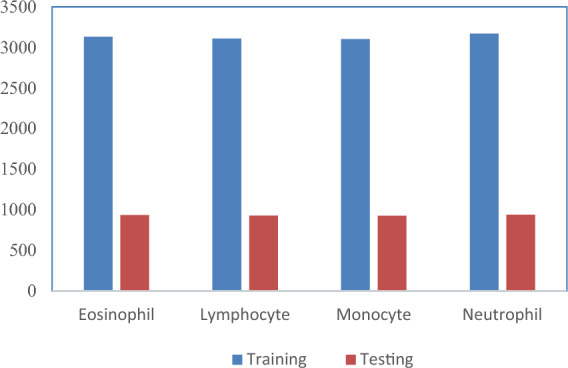
Training and testing image count distribution after the augmentation process.

Sample augmented images of four classes are shown in Fig. 5. A total of 16,249 images were generated across four classes using the augmentation technique, and the distribution is as shown in Fig. 6. Among these, 12,515 images were utilized for training and validation, consisting of 3,133 eosinophil images, 3,109 lymphocyte images, 3,102 monocyte images, and 3,171 neutrophil images. The remaining 3,734 images were reserved for testing, which comprised of 936 eosinophil images, 931 lymphocyte images, 927 monocyte images, and 940 neutrophil images.

### Hyperparameter tuning

The DDRNet models were fine-tuned to enhance their performance by adjusting various hyperparameters, such as the dropout rate, batch size, epochs, learning rate, and optimization units implemented in the gradient. This study used a gradient optimizer named Adam and a dropout factor of 20%. Adam was the best gradient optimizer, and a dropout of 20% of features was found to reduce overfitting to a greater extent, thus improving the impact of generalization in most processes of the DDRNet models. The customized DDRNet model was trained over 30 epochs using the Adam optimizer and a learning rate of 0.01. The learning rate schedule was also thought to be constant. The DDRNet model was trained for 2040 iterations.

### DDRNet model feature analysis

**Figure 7:**
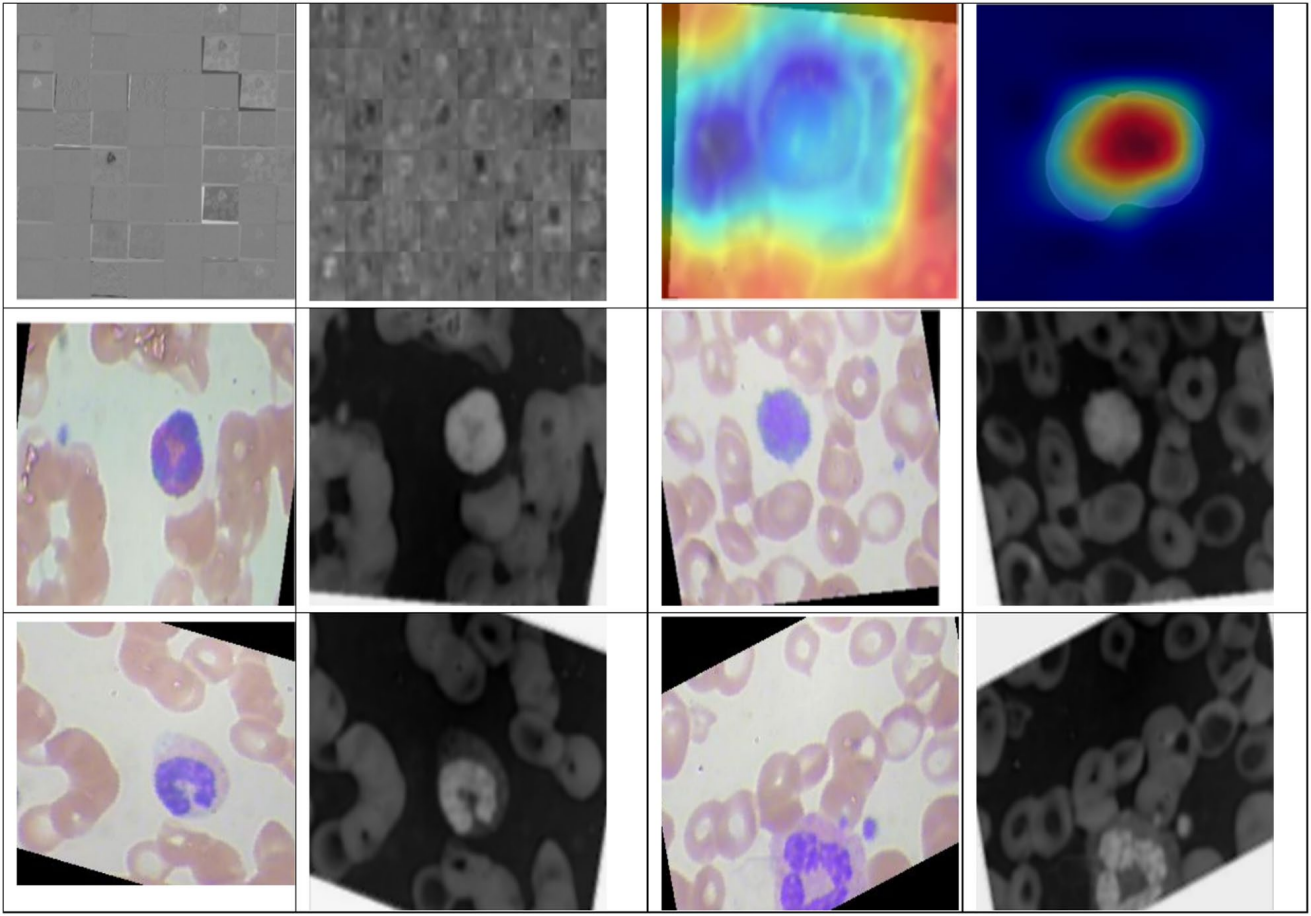
Visualization of the convolution layer features.

Figure 7 describes the features acquired with the help of the inner layers of DDRNet during the training phase. Gradient-weighted Class Activation Mapping (GradCAM) provides insights into the regions of the input images that contributed most to the model’s classification decision. Visualization of features through GradCAM helps clinicians and researchers interpret the decision-making process of DDRNet, offering transparency and aiding in understanding the model’s focus on relevant features. The intermediate layers’ visual feature inspection demonstrates the resilience of retrieved features that retrain the model. This depiction significantly enhances the understanding of how the DDRNet model internally interprets the multiclass blood image. The outcomes of applying 32, 3 × 3 layer1 convolutional filters are visualized in the first image of Fig. 6. The adjacent image shows the results of applying layer six convolution filters. DRDB, GLFEB, and CSAB blocks capture the edges in this instance are more robust than they were in the prior feature image. Further convolution blocks help to extract even more class-specific and inter-class discriminating feature maps from the image. All 64 image variations reflect a distinct feature and contribute to the fully connected convolution layers utilized for the classifications. Figure 7 confirms the exceptional ability of DDRNet to extract features that discriminate images of eosinophil, lymphocyte, monocyte, and neutrophil. The fact that distinct regions are displayed in different colours demonstrates the feature block’s capacity to discriminate between images belonging to the four classes of Leukemia.

### Evaluation metrics

Evaluation measures are essential for evaluating the effectiveness of a trained model. The performance metrics used to evaluate the effectiveness of the DDRNet model included accuracy, precision, recall, F1-score, and confusion matrix. The testing accuracy was ascertained by predicting the result of the DDRNet model on the test data. A confusion matrix was utilized to evaluate the performance of each class in the proposed DDRNet model. To assess the accuracy of testing, the results obtained during the training phase were compared with the test set obtained from the partitioned dataset. Equations (16–19) provide the details of the evaluation metrics:

16 $$Accuracy = \frac{D}{D + E + F + G}$$

17 $$Precision = \frac{D}{D + E}$$

18 $$Recall = \frac{D}{D + W}$$

19 $$F1 Score = \frac{2*Precision*Recall}{{Precision + Recall}}$$

where “D” represents the true positive result, “E” is the true negative result, “F” is the false positive result, and “G” is the false negative result. When the model accurately predicts the positive class, the results are actual positives (D). When the network successfully estimates the negative class, the results are referred to be True Negative (E). A false positive (FP) or false negative (FN) occurs once the model incorrectly estimates the positive class as the negative class or the negative class as the positive class. Table 3 exhibits the evaluation metrics of the four class classifications using the DDRNet Model, including precision, recall, F1 score, and Mathews Correlation Coefficient (MCC).

**Table 3:** Evaluation metrics of the Four class classification using the DDRNet Model.

Class	MCC	Recall	Precision	F1-score	Accuracy
Eosinophil	0.97	0.99	0.99	0.98	91.1
Monocyte	1.00	1.00	1.00	1.00	92.8
Neutrophil	0.97	1.00	0.96	0.98	91
Lymphocyte	1.00	1.00	1.00	1.00	93

The confusion matrix systematically captures and quantifies the model’s predictions across different cell types. Precision, recall, and F1 score metrics were calculated for each cell type to better understand the model’s performance in terms of correct predictions (D and E) and false predictions (F and G). The architecture of DDRNet is built to extract intricate features from input images. This feature enables the model to identify subtle patterns and characteristics that can suggest particular cell types, even when the differences are less pronounced. Augmenting the data through adjusting lighting, rotation, and scaling techniques enhances the model’s capacity to generalize across varied structures. The augmentation of training data diversity proves effective in addressing borderline cases. The utilization of explainability tools aids in identifying the features, which is especially beneficial in situations characterized by ambiguity.

### Model training and validation

**Figure 8:**
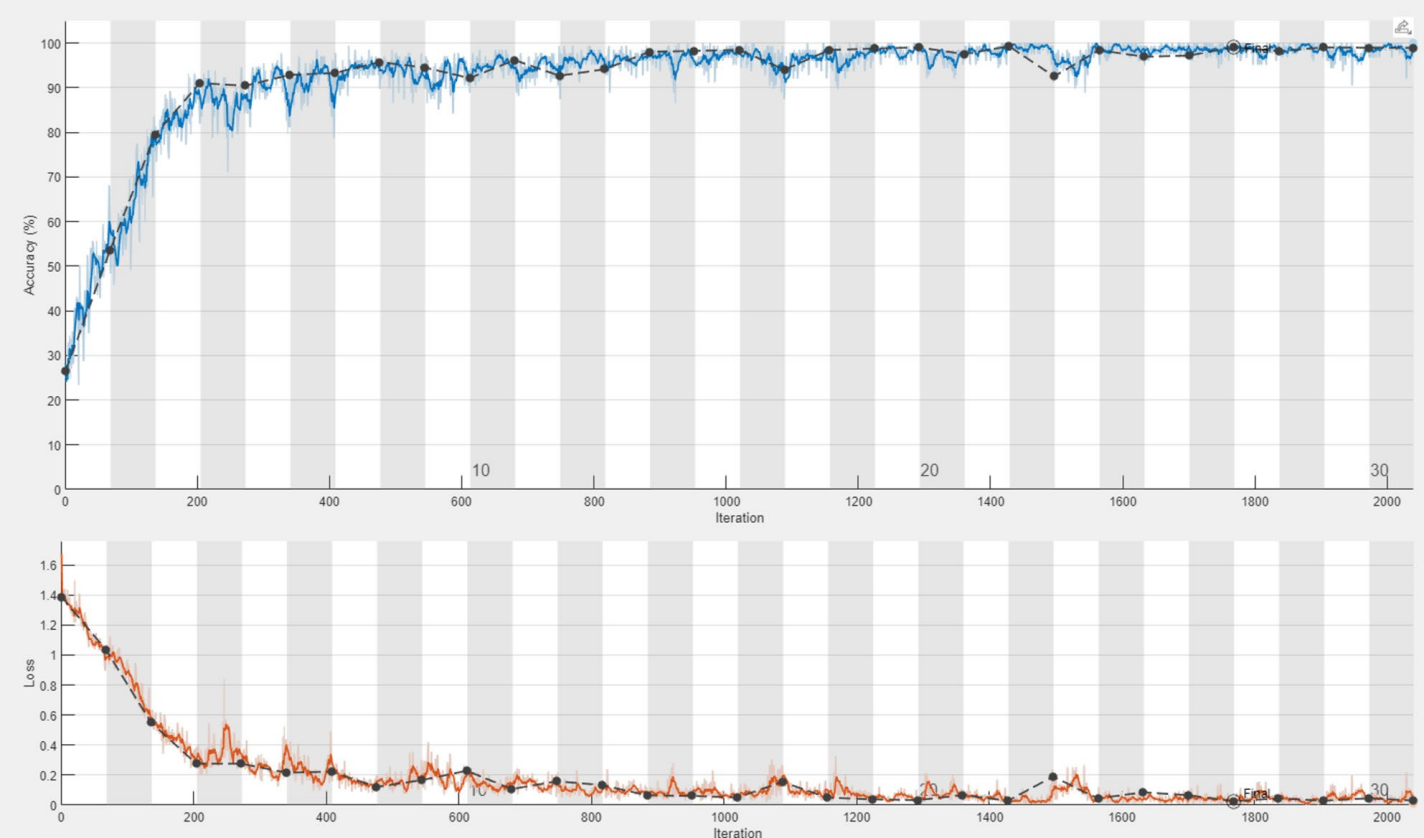
The DDRNet ‘s learning process concerning models’ loss, number of epochs and accuracy .

The classification accuracy of the DDRNet model was evaluated by training and testing it using the augmented data. The model architecture comprises three phases: a deep residual dilated block, followed by blocks that enhance channel and spatial features and global and local feature creation. The ADAM optimizer was employed to train the model over 30 epochs with 2040 iterations. The accuracy and loss curves for each fold of the training and validation sets are illustrated in Fig. 8. The training loss curve decreases as the number of training epochs increases, and similar behavior in the opposing trend is seen for the training accuracy. After 200 iterations, the positive slope’s change rate gradually slows and flattens out. Group normalization was employed during the DDRNet training process, and a learning rate of 0.01 facilitated better and quicker convergence. The model reached saturation more quickly, and the training accuracy improved over time. The DDRNet model incorporates BN and DO techniques to reinforce the stability and efficacy of ALL classification. These methods contribute to sustaining a uniform input distribution across diverse layers and mitigating the risk of overfitting.

Although the validation accuracy increased over time, it changed throughout training. DDRNet has the most straightforward architecture of all the listed custom models. DDRNet is capable of learning a variety of characteristics that are pertinent to microscopic cells. The 2000 iteration of 30 epoch execution time is 161 min 20 s. The architecture of DDRNet is computationally more straightforward, with fewer trainable parameters, and more compact taking less time to train.

**Figure 9:**
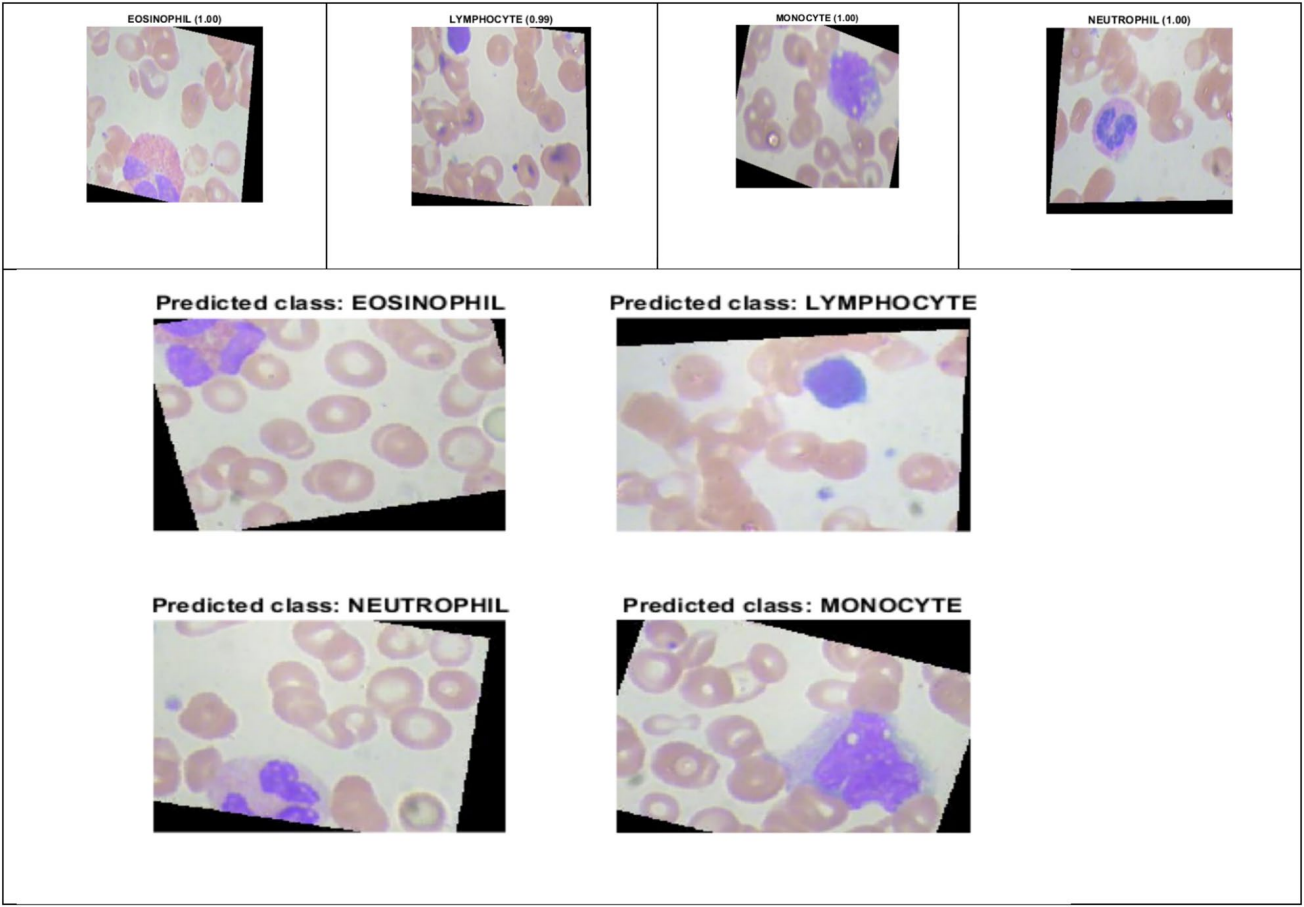
DDRNet Model Prediction Score.

Figure 9 illustrates the prediction score of DDRNet for eosinophil, lymphocyte, monocyte, and neutrophil images. The DDRNet model achieved the highest prediction scores for eosinophil (1.00), monocyte (1.00), neutrophil (1.00) and slightly lower for lymphocyte (0.99). The high prediction score of DDRNet reveals a greater level of confidence in its predicted class. The higher prediction score for eosinophil, monocyte, neutrophil and lymphocyte imply a higher likelihood of ALL disease, and it can aid the clinical decision to proceed with further treatment based on the prediction.

#### Analysis of DDRNet performance

Deep residual dilated block (DRDB) utilized residual connections to improve gradient flow and decreased the depth of the network by dilated convolution network to reduce the model complexity and trainable parameters. Building an accurate and efficient model for multi-class classification is a complex task that demands careful consideration of appropriate features that lower the ambiguity among the eosinophil, lymphocyte, monocyte, and neutrophil images. It is comparatively more challenging than ALL binary classification. In the context of multi-class ALL classification, data augmentation has demonstrated its effectiveness in enhancing the accuracy of DDRNet model. Through the utilization of data augmentation techniques, the DDRNet model developed the ability to adapt to variations in the input images, including variations in orientation, scale, and intensity. As a result, the DDRNet model achieved better generalization, and ultimately, better performance on test data. Furthermore, data augmentation also tackled the issue of imbalanced data in ALL classification. By increasing the samples in the underrepresented class through augmentation, the DDRNet model learned to detect the unique characteristics that differentiate the four classes, resulting in enhanced classification accuracy. The Global and local feature enhancement block (GLFEB) selects the highest activation in each feature map using global average pooling, where the average activation in each feature map is computed. Group normalization (GN) enhanced the performance of DDRNet by reducing the internal covariate shift that arises during the distribution of ALL input images to layer changes during training. Data augmentation and GN increased the robustness to input variations and reduced the sensitivity to hyperparameters such as learning rate and weight decay. GN has been shown to be more effective with small batch sizes to capture the fine-grained details within the eosinophil, lymphocyte, monocyte, and neutrophil images. GLFEB combined global and local features and captured the overall context of ALL images and their specific details, leading to more discriminative and accurate feature representations. DDRNet model captured more complex inter and intra-class patterns and relationships between the features using the CSAB block, leading to better performance.

Tables 4 and 5 illustrate the efficiency of the recommended DDRNet model with various residual networks such as ResNet 18, ResNet 50, and ResNet 101 based on metrics including MCC, recall, precision, F1-score, accuracy, and execution time. The results demonstrate that the recommended model surpasses the other residual networks with regard to accuracy. Moreover, the proposed model takes only 161m19sec for computation, whereas ResNet 18, ResNet 50, and ResNet 101 require 399m17s, 1203m21s, and 1679m39s, respectively, indicating superior computational efficiency. Notably, the proposed model’s computational time is almost 90% less than that of ResNet 101. Moreover, the proposed model achieves an MCC, precision, recall, and F1-score of 1, whereas ResNet 18 and ResNet 101 show variations in performance values.

**Table 4:** Performance Comparison of the proposed DDRNet with Residual Model.

Model	MCC	Recall	Precision	F1-score	Accuracy	Time
ResNet 18	0.97	0.99	0.99	0.98	99.06	399m17s
ResNet 50	1.00	1.00	1.00	1.00	99.20	1203m21s
ResNet 101	0.97	1.00	0.96	0.98	99.60	1678m39s
Proposed DDRNet	1.00	1.00	1.00	1.00	99.86	161m19s

**Table 5:**
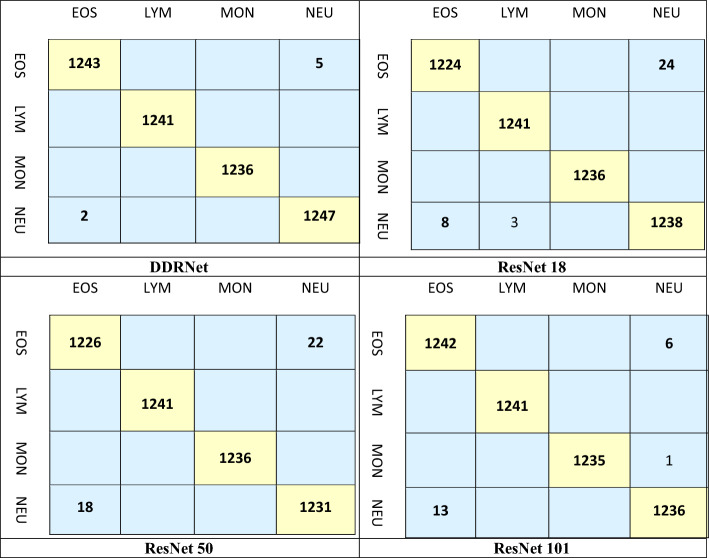
Confusion matrix of the proposed DDRNet and the other existing residual networks.

The confusion matrix generated for the proposed DDRNet is compared with the residual network to show the impacts of the classification of leukemia. The confusion matrices obtained for all the models are shown in Table 5.

Convolutional layers acquire hierarchical features that include local patterns and structures that are improved by translational invariance, enabling the recognition of diverse cell morphologies. The model extracts and learns features at different levels, enabling it to capture both subtle and prominent morphological characteristics associated with each cell type. Augmentation techniques, such as rotation, scaling, and contrast enhancement, are applied during training to introduce variations in cell morphology. This ensures that the model is exposed to a diverse range of morphological features, enhancing its ability to generalize across different cell types. Group normalization stabilizes and normalizes these properties, enabling the model to effectively handle variances in cell morphology across distinct cell types like eosinophils, lymphocytes, monocytes, and neutrophils. The utilization of the deep residual dilated block enables the network to effectively adjust to various spatial scales and accurately capture intricate class-specific features included in the cell images. Dilated convolutions aid in expanding the receptive field, which is essential for detecting patterns at different levels. By allocating varying weights to distinct channels according to their significance, channel attention enables the model to concentrate on the most illuminating characteristics for cell type differentiation. The model highlights crucial regions and discards unnecessary ones with the use of spatial attention. Simultaneously capturing both spatial and channel-wise information, makes it capable of capturing intricate correlations among the data. The attention mechanism has the potential to be useful in identifying tiny details and patterns that differentiate various cell types.

#### Ablation study

**Figure 10:**
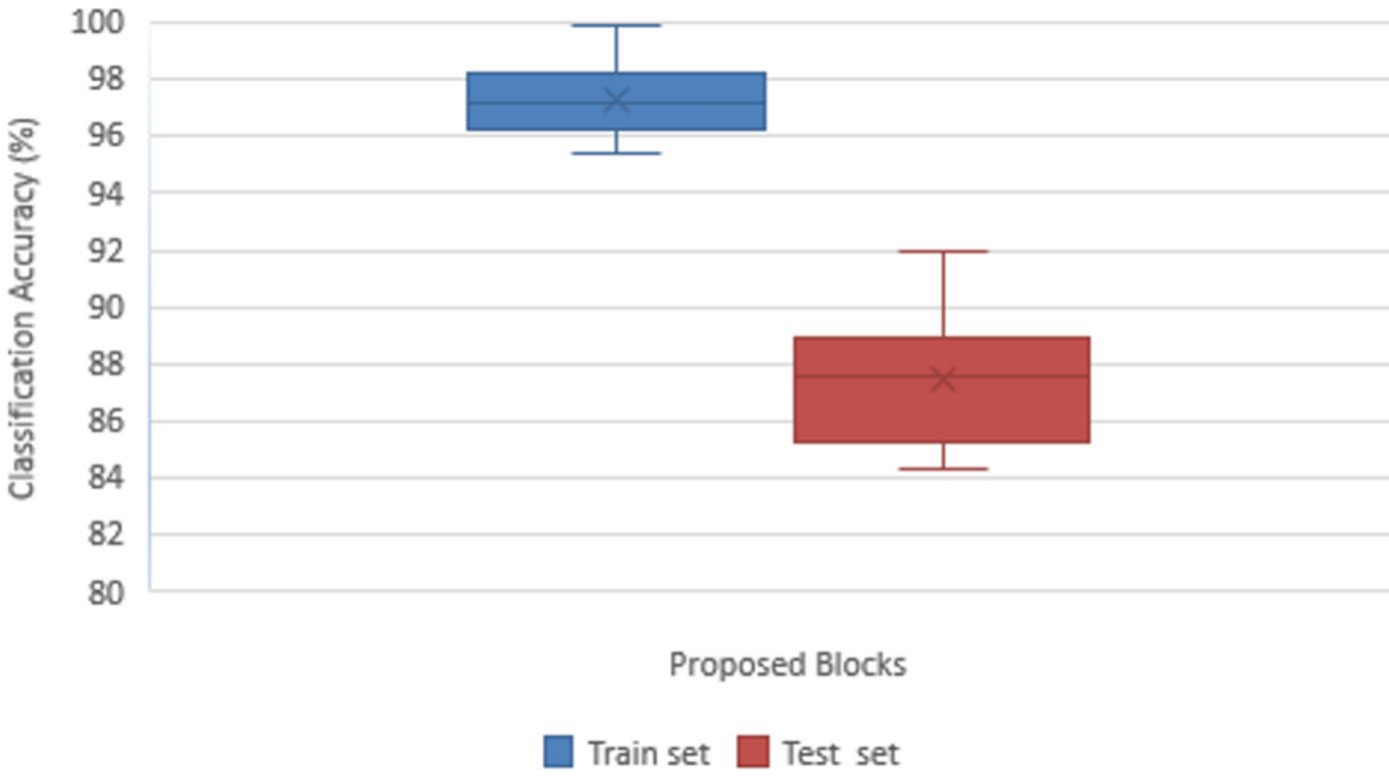
Impact of ablation study on proposed blocks in ALL classification.

The proposed DDRNet model’s various blocks were examined using an ablation study to determine their effects on accurately classifying ALL. It is clear that adding the various blocks, as stated in Table 6, causes the model’s overall performance to improve steadily. Accuracy, Precision, Recall, F1-score, and Confusion matrix of the ALL images are the metrics used to reveal the efficiency of the suggested DDRNet classification architecture. Figure 10 depicts the impact of the ablation study on the suggested blocks for classifying ALL.

**Table 6:** Classification Accuracy of ALL Dataset in Train and Test Set for DDRNet Model.

Proposed modules	Landmark classification accuracy (%)	
	Train set	Test set
Convolution block	95.37	84.30
Convolution + DRDB Block	96.22	85.23
Convolution + DRDB + CSAB Block	96.48	86.14
Convolution + GLFEB Block	97.15	87.56
Convolution + GLFEB + CSAB Block	97.39	87.98
Convolution + DRDB + GLFEB	98.21	88.93
Convolution + DRDB + GLFEB + CSAB Block DDRNet	99.86	91.98

Convolution block is considered the baseline of the proposed model, which attained a classification accuracy of 84.30%, comprising only a convolutional layer followed by a max pooling layer and a group normalization, which normalizes to global features for enhanced classification. Group normalization simultaneously normalizes the channel and batch dimensions by separating and normalizing the channels into groups. Compared to batch normalization, group normalization is less reliant and stable on huge mini-batch sizes, making it advantageous in situations involving smaller batch sizes. Group normalization eliminates the dependence on the mini-batch and batch size sensitivity during the training phase.

The DRDB block is implemented along with the baseline convolution model that attained a classification accuracy of 85.23%. The conventional residual block between the layers retained more original information that extracted the general feature maps for improved accuracy of test data. The generated features are enhanced using CSAB embedded in the Convolution and DRDB block where global averaging and max-pooling are employed, resulting in excellent feature representation and improving accuracy to 86.14%.

Similarly, another study with the GLFEB module is performed with the baseline convolution layer, resulting in a better accuracy of 87.56%. The resultant features are subjected to the channel and attention model for enhanced feature representation, achieving an accuracy of 87.98%. The baseline model is connected with DRDB, GLFEB, and CSAB blocks for testing and training the ALL dataset since both blocks showed improvement in classification accuracy. The internal covariate shift developed during the distribution is minimized through global normalization as the GLFEB finds the highest activation in each feature map. GLFEB generates more accurate and discriminative feature representations by combining global and local features, capturing the overall context of ALL images and the individual details therein. Using the CSAB block, the DDRNet model was able to capture associations and inter- and intra-class patterns more intricately, improving accuracy to 91.98%.

**Figure 11:**
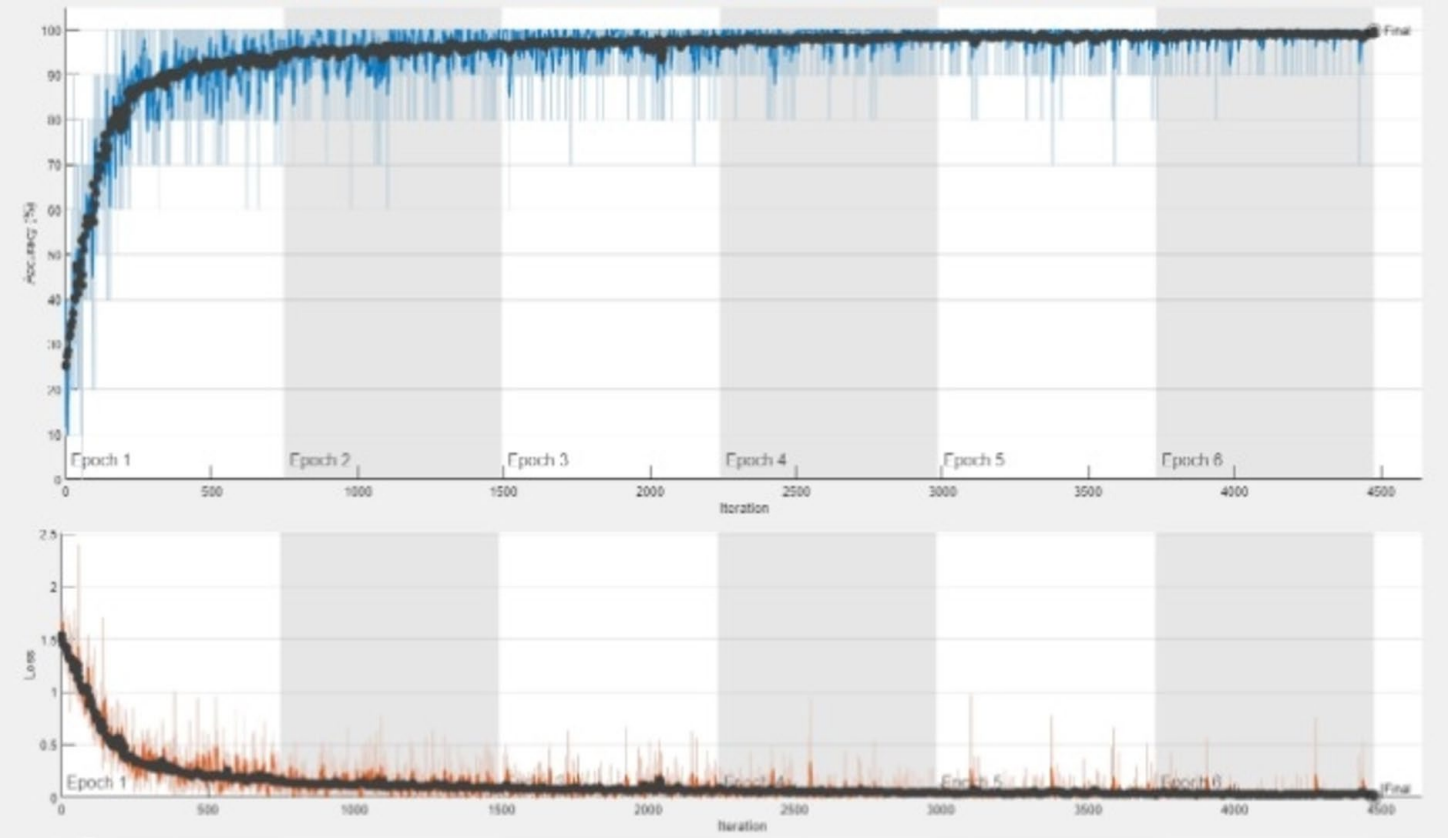
Performance of the proposed model in ALL^49^ dataset.

The proposed DDRNet has been thoroughly trained and assessed using the Acute Lymphoblastic Leukemia (ALL) database^49^ in addition to the multi-class Kaggle dataset^14^. The ALL training data consisted of 73 subjects, including 47 with ALL (7272 cancer cell images) and 26 normal individuals (3389 images), totaling 10,661 cell images. The test set comprised of 2586 cell images.The experimental findings demonstrate that on the ALL dataset, the DDRNet obtained a remarkable accuracy of 99.86% and the training and testing accuracy plot is shown in Fig. 11. This additional validation demonstrates the model’s robustness in handling a variety of data distributions and shows that it can generalize effectively across diverse datasets.

#### Performance comparison with existing leukemia classification research models

Table 7 presents a comparative analysis of the accuracy and efficacy of the DDRNet architecture with cutting-edge models for ALL classification. Karthikeyan et al.^7^ analyzed microscopic images by extracting GLCM features and classifying them using random forest, achieving an accuracy of 90%. Neoh et al.^20^ utilized an intelligent decision support system with machine learning models and achieved an accuracy of 96.72%. Rehman et al.^10^ achieved an accuracy of approximately 97.78% using convolutional neural networks for a dataset from Pakistan. Additionally, YOLOv4 was used to analyze ALLIDB1 and C NMC 2019 datasets and attained an accuracy of 96.06%^32^. The proposed DDRNet model achieved a training accuracy of 99.86% for blood cell dataset and 99.86% accuracy for Leukemia Dataset^49^ with fewer parameters and less computation time compared to the ML and DL approaches.

**Table 7:** Comparison of DDRNet with the state-of-the-art approaches.

Reference no	Dataset	Method	Accuracy (%)	Type
^7^		RF and GLCM	90.00	Machine learning algorithm
^20^		MLP, SVM, and Dempster-Shafer classifiers	96.72	
^10^	Amreek Clinical Laboratory Saidu Sharif Swat KP Pakistan	CNN	97.78	Deep learning techniques
^32^	ALLIDB1 and C NMC 2019	YOLOv4	96.06	
Proposed	Blood Cell Image^14^	DDRNet	99.86	
	Leukemia Dataset(ISBI 2019)^49^		99.86	

## Conclusion

An efficient deep residual layer, known as DDRNet, is proposed in this research for detecting acute lymphoblastic leukemia (ALL) using microscopic images. Underneath the microscope, it is generally challenging to distinguish pre-leukemic cells from normal cells since their morphologies are similar. DDRNet comprises a deep residual dilated block optimized feature Block (DRDB) followed by a global and local feature enhanced block (GLFEB) and a Channel and Spatial Attention Block (CSAB). Without increasing the computation complexity, DRDB block employs residual dilated convolution blocks that allow the information to flow to the adjacent layers and make the training much easier and generalization much better. GLFEA extracts global and local features maps to capture class-specific information. CSAB block extracts features from different channels and exploits spatial features’ inter-class relationships while suppressing irrelevant information. This allows for effective feature learning and extraction, ultimately leading to accurate blood cell classification. The DRDB, CSAB, and GLFEB blocks work together to provide an excellent representation of features for blood cell classification. The results of the ablation experiments provided evidence of the effectiveness of the DRDB, GLFEB, and CSAB blocks in enhancing feature extraction and improving classification accuracy. Furthermore, the comparison with the cutting-edge ResNet models reinforced the efficacy of the DDRNet model. Experimental results validated the exceptional performance of DDRNet in ALL classification, with an impressive F1-score of 96%, high training accuracy of 99.86%, and testing accuracy of 91.98%. Overall, the DDRNet model is a better tool for pathologists and oncologists to use when making clinical choices about leukemia diagnosis. The DDRNet model’s architecture and optimization strategies have been carefully designed to minimize computational overhead while ensuring efficient and effective multi-class blood cell image classification. Our research study suggests that the DDRNet demonstrates promising scalability. The model leverages optimized algorithms and parallel processing techniques to handle substantial workloads without compromising performance.

The proposed DDRNet architecture relies heavily on the availability of high-quality labeled data for training. The model’s performance may suffer, especially when dealing with rare blood cell types when the dataset is limited. Augmented data can enhance the model’s ability to handle variations in blood cell types. Handling unbalanced datasets is a major difficulty in medical image analysis, particularly blood cell classification. Future work could focus on developing techniques to handle imbalanced datasets, such as GAN and other data augmentation techniques or using advanced loss functions that penalize misclassifications of minority classes more heavily. Although the CSAB block enhances interpretability, understanding the exact decision-making process within the model remains challenging. Providing more transparent explanations for its predictions could improve trust and adoption. Continuously refine attention mechanisms (such as CSAB) to strike a balance between feature enhancement and interpretability. Investigate alternative attention mechanisms or adapt them specifically for blood cell classification. The model’s predictive performance and diagnostic accuracy may be improved by incorporating data from other modalities, such as blood cell morphology, immunophenotyping information, and clinical metadata. Future research could look into methods for efficiently combining data from several modalities to increase the precision of blood cell classification and offer more thorough diagnostic insights. Incorporating additional information, such as patient history, may also improve overall accuracy.

Future research could focus on designing novel user interfaces and tools for decision support systems that offer practical insights and promote cooperation between AI systems and medical professionals. The current study focused on the classification of four types of ALL. To expand on this research, future studies can experiment with a diverse dataset that includes various subtypes of leukemia, such as CML, AML and CLL. The robust classification models can be developed by combining deep learning and ensemble learning. In addition, the integration of explainable artificial intelligence methods can provide clinicians with insights into the decision-making process of these models, thus increasing their trust and adoption. Robust classification systems for each subtype can be deployed in clinics for more personalized and effective treatment strategies, ultimately improving patient outcomes.

## Data Availability

The datasets generated and/or analysed during the current study are available in the https://www.kaggle.com/paultimothymooney/blood-cells repository. The ALL Challenge dataset of ISBI 2019 was downloaded from Kaggle^49^.

## Code availability

Programming codes and the data supporting the findings of this study are available upon reasonable request.
